# Supplementary material for: Micro-metric electronic patterning of a topological band structure using a photon beam
Source: Sci Rep. 2015 Nov 6;5:16309. doi: 10.1038/srep16309 (PMC4635360; doi:10.1038/srep16309)
Supplement: Supplementary Information [file srep16309-s1.pdf]

Micro-metric electronic patterning of a topological band structure using a photon beam

(supplementary information [SI])

E. Frantzeskakis[1,*], N. de Jong[1], B. Zwartsenberg[1], Y. Huang[1], T. V. Bay[1], P. Pronk[1], E. van Heumen[1], D. Wu[1], Y. Pan[1], M. Radovic[2,3], N. C. Plumb[2], N. Xu[2], M. Shi[2], A. de Visser[1] and M. S. Golden[1,°]

[1]Van der Waals-Zeeman Institute, Institute of Physics (IoP), University of Amsterdam, Science Park 904, 1098 XH, Amsterdam, The Netherlands
[2]Swiss Light Source, Paul Scherrer Institut, CH-5232 Villigen, Switzerland
[3]SwissFEL, Paul Scherrer Institut, CH-5232 Villigen, Switzerland

SI-1. RESPONSE TO HIGH-FLUX ILLUMINATION
(BSTS1.46: evolution of the energy shift and experimental broadening with increasing photon fluence)

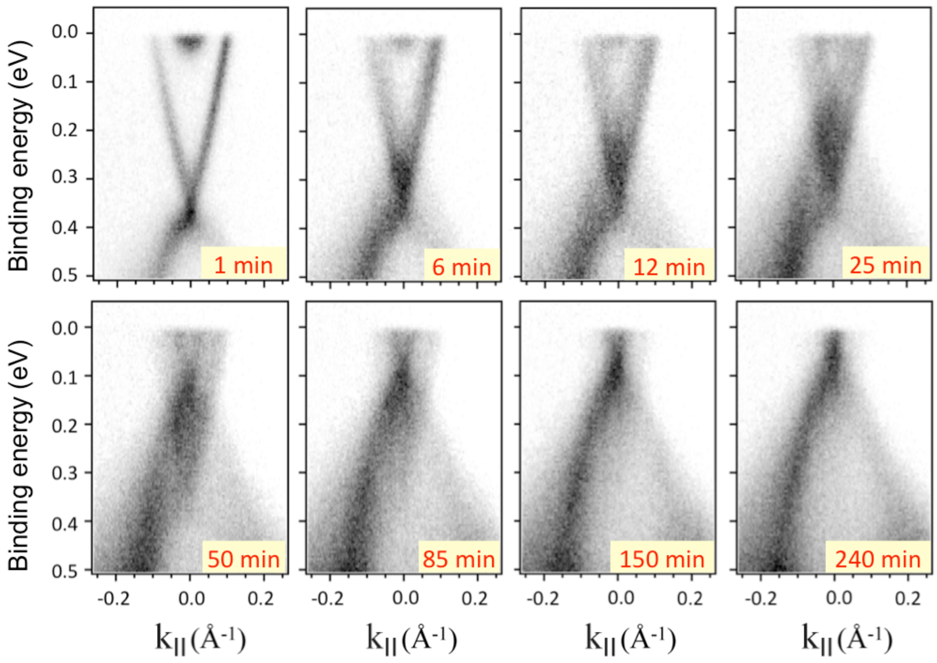

Figure S1: The evolution of the electronic structure of $Bi_{1.46}Sb_{0.54}Te_{1.7}Se_{1.3}$ under high-flux illumination. The electronic band structure of $Bi_{1.46}Sb_{0.54}Te_{1.7}Se_{1.3}$ (BSTS1.46) near the Fermi energy when exposure to high-flux illumination [3.2×10^{21} photons/(s m^2)] gradually increases to 240 min. The total energy shift in this time interval is around 350 meV. The experimental broadening increases in the first 30 min and then it gradually decreases. When all sample locations under the beam spot have reached the saturation energy value, the experimental features are sharp again.

Fig. S1 presents the evolution of the electronic structure of BSTS1.46 as exposure to high-flux [3.2×10^{21} photons/(s m^2)] illumination gradually increases to 240 min. The experimental broadening due to the inhomogeneous beam profile increases during the first 30 min (upper panels). On further exposure, more and more locations under the beam spot reach a saturation energy shift; these timescales mark the

* E.Frantzeskakis@uva.nl
° M.S.Golden@uva.nl

gradual transition from uniform broadening to shadow-like intensity around the dominant energy shift (first two panels of the lower row). When all sample locations under the beam spot reach the maximum energy shift, or in other words flat-band conditions are established throughout the area covered by the photon beam, the experimental features are sharp again (last two panels of the bottom row).

SI-2. BAND STRUCTURE SIMULATIONS AFTER INHOMOGENEOUS ILLUMINATION

The simulation input is the experimental band dispersion before exposure to the high-fluence photon beam. The electronic band structure after high-fluence exposure is simulated by means of a calculated spatial profile of the upward energy shift. As a first step, the intensity profile of the photon beam (I) is approximated by a two-dimensional Gaussian with a FWHM corresponding to the actual beam size used (100 μm × 30 μm). In order to account for different energy shifts within the overall beam footprint, in the simulation the beam is sub-divided into N concentric elliptical rings, each having an area $A = \pi \times 30/100 \times [(n+1)^2 - n^2]$, where n is zero or a positive integer. The photon-induced energy shift is calculated for each ring and each result is weighted by the local beam intensity on the relevant ring and the ring's area. Following our recent study **[S1]**, we have used a double-exponential function to relate the photon-induced energy shift and the photon fluence (or equivalently exposure time)

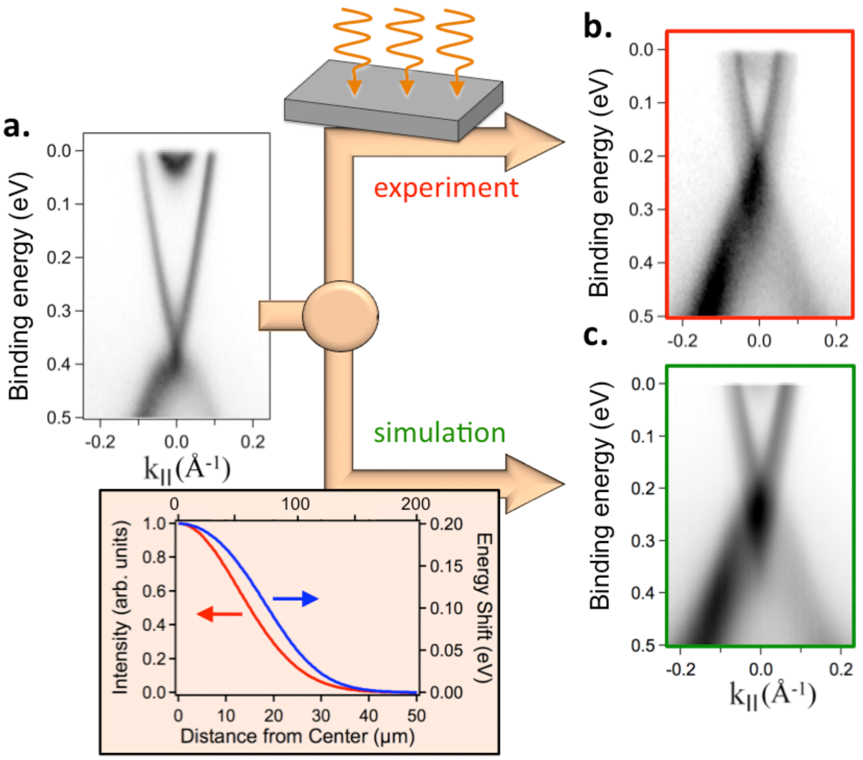

Figure S2: The effect of inhomogeneous illumination on BSTS captured by simulations of the experimental data. (a) The near-E_F electronic band structure of $Bi_{1.5}Sb_{0.5}Te_{1.7}Se_{1.3}$ (BSTS1.5) with downward band bending. (b) The near-E_F electronic band structure of BSTS1.5 after exposure to a photon flux of 1.3×10^{21} photons/(s m^2) for 60 minutes. (c) Simulation of the experimental dispersion presented in panel (b) by considering a Gaussian intensity profile for the writing photon beam. The local energy shift is a function of the local intensity within the inhomogeneous beam spot (see section SI-2). For the case before saturation of the photon-induced band flattening, the total ARPES spectrum measured is thus a sum over regions with different local energy shifts, an approach which reproduces the experimental results.

Fig. S2a shows the near-E_F electronic structure of $Bi_{1.5}Sb_{0.5}Te_{1.7}Se_{1.3}$ (BSTS1.5), a quaternary bulk insulating TI. The energy dispersion corresponds to essentially saturated band bending conditions, arrived at by exposure to UHV for a few hours. Exposure to a high-fluence EUV beam [1.3×10^{21} photons/(s m^2) for 60min] results in an upward energy shift of approximately 130 meV accompanied by

significant spectral broadening as can be seen in Fig. S2b. The resulting electronic structure has also been simulated in Fig. S2c. The simulation shown has been obtained using characteristic exponential time constants (tau1, tau2) = (0.6min, 14.8min), values as determined in Ref. S1 for an identical photon flux. We note that the basic ability of the simulation to reproduce the experimental broadening does not depend on the precise form of this function, as long as it adequately describes an energy shift that slows down with increasing time (or fluence), until saturation is obtained. In this case, both the energy shift and the experimental broadening are well captured by the model. The simulated dispersion is then calculated according to:

$$Output(E) = \sum_{n=0}^{N} Input(E + \Delta E_{h\nu}) \times I \times A \times f_{FD(E)} \qquad \text{Eqn. [S1]}$$

with I and A defined previously, $\Delta E_{h\nu}$ representing the photon-induced energy shift and $f_{FD(E)}$ representing the Fermi Dirac distribution at the temperature of measurement (17K). The diagram under panel (a) of Fig. S1 represents the spatial profile of the beam intensity (left axis, red curve) and the resulting spatial profile of the energy shift (right axis, blue curve) along the small axis (bottom scale) and along the large axis (top scale) of an elliptical beam spot with a FWHM of 100 μm × 30 μm.

SI-3. RESPONSE TO HIGH-FLUX ILLUMINATION (BSTS1.46 vs. Bi_2Se_3)

Fig. S3 compares the effect of high-flux illumination on BSTS and Bi_2Se_3. The left panels show the electronic band structure of Bi_2Se_3 (a) and BSTS1.46 (c) after long exposure to residual gases, at locations which were not previously illuminated. In both cases the band bending induced by residual gas adsorption is on the verge of temporal saturation. For Bi_2Se_3 one observes clear quantum well states both in the valence and conduction band. The lower quantum well states exhibits Rashba-Bychkov spin-split branches in agreement with previous reports **[S3-S5]**. Band bending of BSTS1.46 on exposure to residual gases saturates before the evolution of multiple quantum well states. In agreement with Fig. 1 of the main text, the conduction band shifts below E_F and gives rise to a single parabolic state.

Both samples have been subsequently exposed to high-flux [3.2×10^{21} photons/(s m^2)] super band gap illumination for 30 minutes. There are major differences in their response. Panel (b) shows that the electronic band structure of Bi_2Se_3 shifts to lower binding energies by approximately 30 meV, with the Rashba-Bychkov splitting of the lowest quantum well state decreasing beyond experimental resolution. These are clear effects of a moderate band flattening and a consequent decrease of the electric potential gradient in the *z*-direction. The same photon fluence is enough to induce much more pronounced changes in the electronic band structure of band-bent BSTS1.46. Panel (d) of Fig. S3 shows that the electronic dispersion is significantly broadened, pointing towards a superposition of spectra shifted in energy with respect to each other. As explained in sections SI-1 and SI-2, we attribute the energy differences between different locations to the inhomogeneous profile of the photon beam, which in turn results in differing photon exposure between areas located in the center and under the wings of the beam spot. Such broadening can be decreased by modifying the time of exposure (e.g. as done for the data shown in Fig. 1 of the main text) or - in principle - by altering the spatial intensity profile of the excitation source. Broadening in the I(E,*k*) images is a signature of the local character of the changes generated by illumination and suggests that photon-induced carriers in the near-surface region of BSTS diffuse at much lower rates than in Bi_2Se_3, as the latter (panel [b]) exhibits no broadening after exposure to the same amount of photons. We therefore observe a direct link between the low bulk conductivity and the slow in-plane diffusion of photon-induced carriers in BSTS. In line with the different diffusion rate of induced carriers, the electronic band structure of BSTS1.46 exhibits a larger energy shift on illumination. A shift of 140 meV (on average) can be inferred by comparison of panels (c) and (d) of Fig. S3. Concluding this

section: more pronounced energy shifts and slower diffusion of photon-induced carriers suggest that bulk-insulating BSTS has a higher potential as a platform for micro-patterning than conventional Bi_2Se_3.

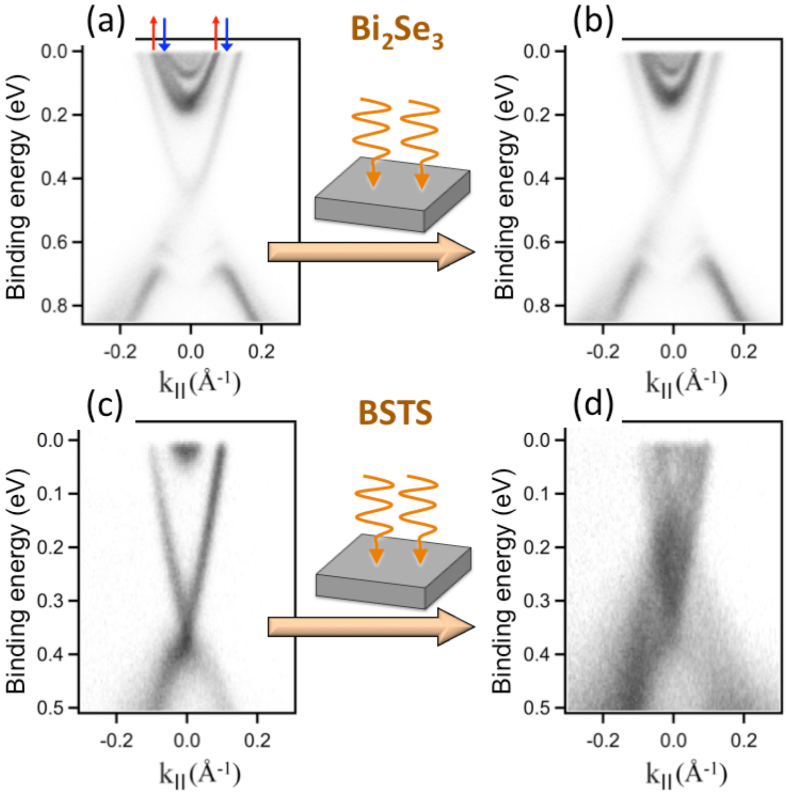

Figure S3: The effect of high-flux illumination on Bi_2Se_3 and $Bi_{1.46}Sb_{0.54}Te_{1.7}Se_{1.3}$. The near-$E_F$ electronic structure of (a) Bi_2Se_3 and (c) $Bi_{1.46}Sb_{0.54}Te_{1.7}Se_{1.3}$ (BSTS1.46) on the verge of maximum band bending through exposure to residual gases under UHV conditions. The bottom of the conduction band is below E_F for both Bi_2Se_3 and BSTS under these conditions. The conduction band of Bi_2Se_3 splits into a series of quantum well states (QWS), the lower of which exhibits Rashba-Bychkov splitting as pointed out by the blue and red arrows. Changes in the near-E_F electronic structure of (b) Bi_2Se_3 and (d) BSTS1.46 after exposure to high-flux illumination [3.2×10^{21} photons/(s m^2)] for 30 min. The energy shift and the spectral broadening are significantly greater for BSTS1.46 than for Bi_2Se_3.

SI - references

[S1] E. Frantzeskakis *et al., Dirac states with knobs on: interplay of external parameters and the surface electronic properties of 3D topological insulators.* Phys. Rev. B **91**, 205134 (2015)

[S2] A. A. Kordyuk *et al., Photoemission-induced gating of topological insulator.* Phys. Rev. B. **83**, 081303 (2011)

[S3] P. D. C. King *et al., Tunable Rashba spin splitting of a two-dimensional electron gas in Bi_2Se_3.* Phys. Rev. Lett. **107**, 096802 (2011)

[S4] Z.-H. Zhu *et al., Rashba spin-splitting control at the surface of the topological insulator Bi_2Se_3.* Phys. Rev. Lett. **107**, 186405 (2011)

[S5] M. S. Bahramy *et al., Emergent quantum confinement at topological insulator surfaces.* Nature Commun. **3**, 1159 (2012)
